# A term infant with severe hypereosinophilia secondary to CMV infection and the *STAT1* gene mutation: a case report: List of authors

**DOI:** 10.1186/s12887-024-04846-4

**Published:** 2024-06-26

**Authors:** Shaimaa Salah, Saleh Nouh Alshanbari, Hassan Musa Masmali

**Affiliations:** 1 Pediatric Department, Faculty of Medicine, Kafrelsheikh University, Kafr El-Shaikh, Egypt; 2 Maternity and Children Hospital, Makkah, Saudi Arabia

**Keywords:** Hypereosinophilia, Cytomegalovirus, *STAT1*, Gene mutation, Infant

## Abstract

Hypereosinophilia is a rare presentation in all age groups, particularly when it is severe, persistent, and progressive. We describe the clinical characteristics and course of severe hypereosinophilia in a full-term Saudi female neonate. A febrile respiratory illness evolved with a progressive increase in peripheral blood leukocyte and eosinophil counts, reaching 44.9% of leukocytes and an absolute value of 57,000 cells/µl. Different etiological examinations (for viral, bacterial, immunodeficiency, hyper IgE syndrome, gene mutations) revealed extremely high CMV antigenemia and a homozygous mutation in the *STAT1* gene. Anhelation was relieved by oxygen and anti-viral treatment. Steroids brought a dramatic response in peripheral blood counts within 24 h. After a 6-week course of antiviral and steroid treatment at home, she had an excellent general condition. Conclusion: Although a rare pathology, it is important to consider genetic disorders when there is an atypical immune response to viral infections.

## Background

Although, mild to moderate hypereosinophilia (HE) is reported in 14-76% of preterm neonates, severe HE in neonates is rare, which favors diagnostic and therapeutic concerns. HE is defined as an absolute eosinophil count (Ec) in neonatal peripheral blood > 500 cells/µl [1]. The condition is classified as mild, moderate, or severe; (mild, Ec – 500–1500 cells/µl; moderate, 1500–5000 cells µl; severe, > 5000 cells/µl). Germline mutations in signal transducer and activator of transcription1 (*STAT1*) gene lead to primary immunodeficiency, they are classified as defects in intrinsic and innate immunity. Homozygous and heterozygous, gain-of-function (GOF) and loss-of-function (LOF) mutations in the *STAT1* gene are described. Here we report an infant with LOF *STAT1* gene mutation and moderate HE that progressed to severe form over 2 months and review the relevant literature.

## Case report

### Clinical data and results

A full-term female of a 2-week of age was incubated for a febrile illness and poor general condition at the Maternity and Children Hospital, Makkah, Saudi Arabia. An uneventful pregnancy and delivery except for a febrile illness in mid-pregnancy was reported, birth weight was 2800 g. She is the firstborn baby in a consanguineous marriage, with no reported family history of immunodeficiency, autoimmunity, mycobacterial infection, prenatal abnormalities, or abortions.

### Clinical findings & diagnostic assessment

**Fig. 1:**
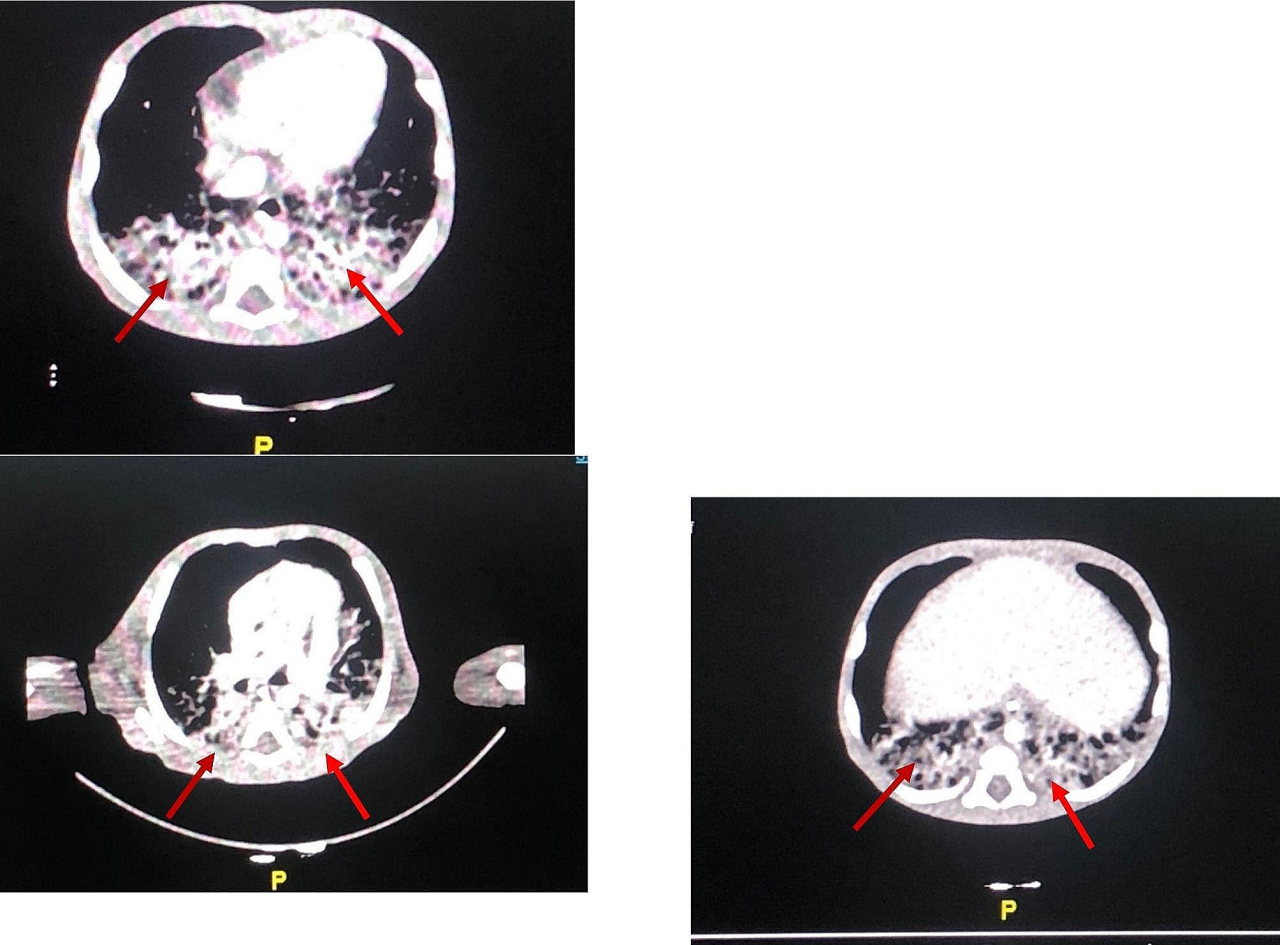
Chest computed tomography. Diffuse bilateral air space consolidation with air bronchogram seen more at the basal segment of both lower lung lobes

At the incubator, a diagnosis of aseptic meningitis was adopted having cerebrospinal fluid (CSF) analysis showing an increased white blood cell count (WBC) – 62/µl; mononuclear – 96.3% and polymorphonuclear – 3.7%, high protein content – 125 mg/dl, and low glucose – 2 mmol/l. CSF cultures didn’t grow organisms. Complete blood count (CBC) showed 21,800 cells/µl leukocytes, 11,800 cells /µl absolute neutrophil count (ANC), 5400 cells/µl Lymphocytes, 1600 cells/µl eosinophils, 13 gm/dl hemoglobin (Hb), and 630,000 cells/µl platelets, erythrocyte sedimentation rate (ESR) was 7 mm/h. Antibiotics in the form of ampicillin and gentamicin and supportive treatment were introduced. She was discharged 19 days later with a satisfactory evolution and a WBC – 22,000 cells/µl, and Ec – 5200 cells/µl. Two weeks later, the infant exhibited frequent high-grade fever and spasmodic cough for which she was admitted to the general pediatric ward. No history of antibiotics or nonsteroidal anti-inflammatory drug use or other premedication was registered. Signs were as follows: blood pressure 95/59 mmHg, heart rate 145/min, respiratory rate 32/min, temperature 39.8 °C, oxygen saturation in room air – 97%, capillary refill time of 2 s, and body weight – 3.5 kg. Occasional desaturation to 80s% was documented with bottle-feeding or coughing. The physical examination was unremarkable except for hepatomegaly of 3–4 cm below the costal margin. No heart murmur or abnormal breath sounds were observed. Initially, the patient was treated for sepsis after sending blood and CSF samples for culture/sensitivity with antibiotics in the form of meropenem and vancomycin. No bacterial growth was revealed. A chest x-ray was performed, which revealed ground glass opacity bilaterally. A differential diagnosis (DD) of a congenital infection versus acquired chlamydia infection was adopted. The striking issue was the steady increase in WBC/Ec reaching 127/ 57,000 cells/µl (44.9%) associated with progressive normocytic anemia (Hb 7.4 gm/dl). The peripheral blood smear showed leukocytosis with striking eosinophilia with no blasts. To reach a specific diagnosis, further tests were performed with the following results: immunoglobulins [IgA, 71 mg/dl (reference range (rr) 70–400 mg/dl); IgG, 850 mg/dl (rr 700–1600 mg/dl); IgE, 204 mg/dl (rr 0-100 mg/dl); IgM, 130 mg/dl (rr 40–230 mg/dl)]. Both renal and liver functions were normal, as well as serum electrolytes, lactate dehydrogenase (LDH) was 755 IU/L. A computed tomography (CT) scan of the chest showed bilateral lower lobe consolidation (Fig. 1). A virology study showed no reactivity to human immunodeficiency virus (HIV) or hepatitis viruses, however, both IgG and IgM to cytomegalovirus (CMV) were high, and CMV polymerase chain reaction (PCR) was sent. Bone marrow aspiration/biopsy was not done. The maternal CMV antibody status was sought however, samples were lost. The possibility of genetically related disease was considered, and whole exome sequencing (WES) was performed.

## Therapeutic interventions

Waiting for the results, treatment was started with intravenous immunoglobulins (IVIG) – 2 gm/kg with no measurable effect. Then, intravenous (IV) prednisone 1 mg/kg per dose every 12 h was initiated. The next day, WBC/Ec came as 62/39,000 cells/µl (29.5%). Fevers finally subsided with the improved general condition. CMV viral load was shown to be – 18,700,000 IU/ml confirming the diagnosis of CMV infection. A CT brain scan, hearing, and ophthalmological assessment were unremarkable. Anti-viral treatment in the form of IV ganciclovir was started at the hospital for 21 days in addition to steroids.

## Follow-up & outcomes

The infant responded favorably to treatment and was discharged later with clear lungs and full oral feeding. CBC before discharge showed WBC/Ec – 17,000/646 cells/µl. Steroid and anti-viral treatment continued at home orally, until clinical and laboratory remission. The result of WES came later with the finding of – a homozygous LOF variant in the *STAT1* gene; c.1760_1761del (p.Glu587fs).

## Discussion

Hypereosinophilia is classified into a primary form; hypereosinophilic syndrome (HES); related to bone marrow proliferative diseases, and a secondary form most commonly related to infections and allergic reactions. HES is defined by a persistent Ec ≥ 1500 cells/µl, absence of a secondary cause, and evidence of eosinophil-associated pathology. While primary HE in children is rare, the secondary (reactive) form is most frequent in this age group [2, 3]. Secondary HE is reported in a wide range of conditions including allergic drug reactions, primary immunodeficiency disorders, connective tissue disorders, respiratory eosinophils (e.g. Churg-Strauss syndrome), aspergillosis, gastrointestinal tract eosinophilia and, paraneoplastic syndromes [1, 4–6]. Hernández-Benítez R et al. reported an infant with severe atopic dermatitis and WBC/Ec – 43,900/30,200 cells/µl [7].

Eosinophils have a vital share in the immune defense mechanisms, besides tissue regeneration and remodeling processes [3]. Through degranulation and the production of a network – the so-called eosinophil extracellular traps (EET), they are believed to have an important share in the pathogen immune response [8, 9]. Being stimulated, eosinophils release inflammatory mediators and procoagulants, which may be associated with thrombotic events and tissue fibrosis [1, 2, 5]. The subsequent organ damage depends on the site of eosinophilic infiltration, which most often affects the skin, circulation, respiratory system, gastrointestinal (GI) tract, and nervous system [10]. Despite children commonly presenting with GI symptoms, contrary to adults, the patient in this report exhibited mainly respiratory symptoms. Similar presenting symptoms were reported by Juan He et al. in a term infant with Ec – 27,000 cells/µl that declined over 3 months with non-specific treatment being of an unclear etiology [11]. Krous HF et al. reported one case of neonatal death due to eosinophilic myocarditis [12].

Often, HE is discovered incidentally. Since the first admission of the present case, eosinophilia was evident in the CBC but most likely passed unnoticed. It wasn’t until the second admission that the severe and progressive increase in WBC/Ec drew attention.

CMV has a broad infectivity range, it is typically associated with immunocompromised patients [13]. There are few documented cases of primary CMV infection associated with eosinophilia [14–17], although it is not uncommon for viral infections to be associated with eosinophilia, however, their antiviral abilities have been demonstrated mainly in respiratory syncytial virus (RSV) studies [18]. They interact with T-cells and antigen-presenting cells via their granules contributing to adaptive immunity [19, 20]. Kobayashi A reported an infant with severe HE; WBC/Ec – 39.8/21.5 × 10^3^ cells/µl associated with reactivation of CMV and drug hypersensitivity evidenced by lymphocyte transformation test (LTT) [21]. Like the case in this report, they documented a rapid improvement of hypereosinophilia and its associated manifestations with systemic corticosteroid therapy, attributing the therapeutic response and transient clinical course to organ failure with hypereosinophilia.

Further areas of research could aim to discover why a remarkably small percentage of CMV-infected patients have been reported to display eosinophilia. Perhaps it is due to a unique response to viral infection by these individuals’ T-cells and eosinophil proliferation signaling; hypothetically, eosinophilia could be explained by these individuals’ recruitment of CD4 T-cells and subsequent production of interleukin 5(IL 5), an “eosinophil colony-stimulating factor” [22]. If so, perhaps these patients’ unique immune systems may also explain why their course of infection is more severe compared to their typical immunocompetent counterparts. In addition, a genetic basis could be suggested based on the findings in the present case. The *STAT1* gene is known to mediate the actions of cytokines involved in innate and adaptive immune defense against viruses and intracellular bacteria [23]. *STAT1* mutated patients either in a dominant or a recessive fashion are susceptible to infections with these organisms which reflects the failure of interferon (IFN)-γ- and IFN-α/β-mediated immunity [24–26]. According to O’Shea JJ et al. *STAT1* mutations probably have a marked impact on the host defense against infections through disturbed T-helper cell responses [27]. *STAT1* gain-of-function (GOF) mutations are described in patients with early-onset chronic mucocutaneus candidiasis, bacterial respiratory tract infections, and humoral immunodeficiency, while loss-of-function mutations are to be considered in patients with early-onset mycobacterial disease, osteomyelitis, respiratory tract infections, and Herpesviridae infection. Baris S et al. reported two patients with autosomal dominant mutations in the *STAT1* gene; presented with oral candidiasis and recurrent CMV infections, in addition to cavitary mycobacterial lung disease early in life (2–4 months of age) [28].The variant in this report is previously described as a pathogenic LOF mutation, inherited in an autosomal recessive fashion, that leads to a clinical entity called immunodeficiency 31B associated with increased susceptibility to mycobacterial and viral infections. This was described in two unrelated infants who suffered from mycobacterial disease and both died of disseminated viral disease [25]. The clinical course introduced in this report can be explained by the detected gene mutation and its impact on the unique immune response to CMV infection manifesting as severe reactive HE and bilateral pulmonary consolidation. In conclusion, we report an infant case with severe hypereosinophilia and systemic symptoms associated with cytomegalovirus infection and *STAT1* gene mutation, and our case might provide novel insight regarding the pathogenesis of reactive HE through a possible link between gene mutation and primary immunodeficiency leading to eosinophil production secondary to imbalance in T-cell stimulation.

## Data Availability

The datasets used in the current report are available from the corresponding author upon reasonable request.

## Abbreviations

°C: Degree Celsius
µl: Microlitre
ANC: Absolute Neutrophil Count
CBC: Complete Blood Count
Cm: Centimeter
CMV: Cytomegalovirus
CSF: Cerebrospinal fluid
CT: Computed Tomography
DD: Differential Diagnosis
Dl: Decilitre
Ec: Eosinophil count
EET: Eosinophil Extracellular Traps
G: Gram
GI: Gastrointestinal
GOF: Gain-of-function
H: Hour
Hb: Hemoglobin
HE: Hypereosinophilia
HES: Hypereosinophilic Syndrome
HIV: Human Immunodeficiency Virus
IFN: Interferon
IgA: Immunoglobulin A
IgE: Immunoglobulin E
IgG: Immunoglobulin G
IgM: Immunoglobulin M
IL 5: Interleukin 5
IU: International Unit
IV: Intravenous
IVIG: Intravenous Immunoglobulins
Kg: kilogram
l: litre
L: Litre
LDH: Lactate Dehydrogenase
LOF: Loss-of-Function
LTT: Lymphocyte Transformation Test
Mg: Milligram
Min: Minute
Ml: Milliliter
mmHg: Millimetres of Mercury
mmol: millimole
PCR: Polymerase Chain Reaction
rr: reference range
RSV: Respiratory Syncital Virus
S: Second
STAT 1: Signal Transducer and Activator of Transcription 1
WBC: White Blood Cells
WES: Whole Exome Sequencing
